# Cell type annotation for scATAC-seq via DNA large language model and graph domain adaptation

**DOI:** 10.1371/journal.pcbi.1014226

**Published:** 2026-04-30

**Authors:** Yan Liu, Sheng Guan, He Yan, Long-Chen Shen, Ji-Peng Qiang, Guo Wei

**Affiliations:** 1 School of Information and Artificial Intelligence, Yangzhou University, Yangzhou, Jiangsu, China; 2 College of Information Science and Technology & Artificial Intelligence, Nanjing Forestry University, Nanjing, Jiangsu, China; 3 School of Computer Science and Engineering, Nanjing University of Science and Technology, Nanjing, Jiangsu, China; 4 School of Computer Science and Information Engineering, Bengbu University, Bengbu, Anhui, China; The Pennsylvania State University, UNITED STATES OF AMERICA

## Abstract

Single-cell ATAC-seq (scATAC-seq) enables the exploration of chromatin accessibility at single-cell resolution, offering critical insights into gene regulation. Accurate cell type annotation is a fundamental prerequisite in scATAC-seq analysis. While cross-modality annotation methods leverage scRNA-seq data for label transfer, they often suffer from modality mismatch and signal distortion. Intra-modality annotation, which utilizes only scATAC-seq reference data, has gained attention for its biological consistency. However, existing methods are limited by insufficient sequence representation and lack of neighborhood modeling during domain adaptation. To address these limitations, we propose scLLMDA, a novel framework for scATAC-seq cell type annotation via DNA large language model and graph-based domain adaptation (GDA). scLLMDA uses a pretrained DNA-specific language model to generate contextual embeddings of peak sequences, which are then integrated with accessibility information to represent individual cells. We construct similarity-based cell graphs for both source and target datasets, and apply a graph neural network to align domains while preserving local structural context. Our approach captures rich sequence semantics and neighborhood dependencies, enabling more accurate and robust cell type annotation across datasets. Extensive experiments on multiple benchmarks demonstrate that scLLMDA outperforms existing methods in accuracy. The source code and implementation of scLLMDA are publicly available at: https://github.com/sheng-guan-2001/scLLMDA.

## Author summary

Single-cell ATAC-seq provides unique insights into gene regulation, but accurate cell type annotation remains challenging. Existing approaches often suffer from modality mismatch or limited feature representation. We introduce scLLMDA, a framework that combines a DNA large language model with graph-based domain adaptation. By capturing sequence semantics and neighborhood structure, scLLMDA achieves more accurate and robust cell type annotation across datasets.

## Introduction

scATAC-seq has emerged as a powerful technique for profiling chromatin accessibility at single-cell resolution [1], offering unique insights into gene regulatory landscapes across diverse cell types [2]. A key step in scATAC-seq data analysis is accurate cell type annotation, which enables downstream analyses such as trajectory inference, differential accessibility analysis, and integration with other single-cell omics datasets. However, due to the inherent sparsity and noise in scATAC-seq data, automated and robust annotation remains a significant challenge.

Existing cell type annotation methods for scATAC-seq can be broadly categorized into two groups based on the omics modality of the reference dataset: (1) Cross-modality annotation [3–5], which involves transferring cell type labels from scRNA-seq or other omics modalities through data integration techniques, has emerged as a widely used strategy in single-cell analysis. However, substantial challenges remain due to inherent differences in data structure, sparsity, and modality-specific biases, which complicate accurate alignment across omics layers. (2) Intra-modality annotation [6], which involves using well-annotated scATAC-seq datasets as references to directly annotate cells from new scATAC-seq experiments, has gained traction as a modality-specific alternative to cross-omics approaches. Several representative methods illustrate the diversity of strategies in this domain. For example, Cellcano [6] uses a two-stage supervised learning approach: an MLP [7] is first trained on reference data to predict target labels, followed by self-distillation on high-confidence “anchor” cells to refine predictions. scATAnno [8] extracts embeddings via SnapATAC2 [9], aligns reference and query data using Harmony [10], and assigns labels through a KNN classifier [11]. EpiAnno [12] adopts a Bayesian neural network [13] to incorporate uncertainty estimation, enhancing robustness and classification accuracy in cell type annotation. With the increasing availability of high-quality manually annotated scATAC-seq datasets, intra-modality annotation has gained more attention due to its reduced modality gap and improved biological consistency. In this work, we focus on the intra-modality scATAC-seq cell type annotation task, where the goal is to assign cell labels to unannotated cells using only scATAC-seq reference data.

Despite recent progress, current intra-modality annotation methods suffer from two main limitations: (1) Limited Sequence Representation: Most methods rely on convolutional neural networks (CNNs) to extract features from DNA sequences of accessible regions [14,15]. However, CNNs are constrained by fixed receptive fields and lack the ability to capture long-range dependencies or contextual biological signals embedded in the genomic sequence. This restricts the richness of the learned representations and may compromise annotation accuracy. (2) Neglect of Neighborhood Context in Domain Adaptation [16]: When adapting knowledge from a labeled source dataset to an unlabeled target dataset, current domain adaptation strategies typically align global feature distributions without explicitly modeling the graph structure or local neighborhood relationships between cells. This can lead to suboptimal domain alignment, especially in heterogeneous or complex cell populations.

**Fig 1:**
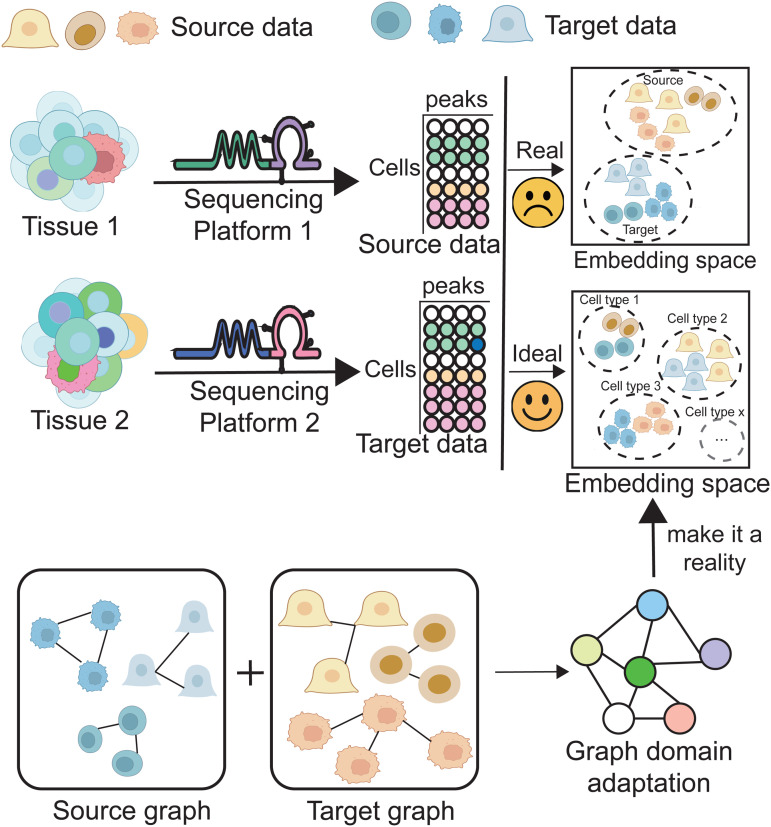
Overview of batch effect and graph-based domain adaptation in scATAC-seq cell type annotation. Cells from different tissues are sequenced using different platforms, resulting in source and target datasets with potential batch effects. The observed embedding space (top right) often separates cells by batch rather than by biological identity, due to sequencing-induced biases. The ideal scenario should organize cells according to true cell types, regardless of platform or batch. To achieve this, we construct source and target graphs based on intra-dataset similarity, and apply a graph domain adaptation strategy to align the two domains. This framework enables batch-aware alignment and improves cross-platform cell type annotation accuracy.

To address these challenges, we propose a novel framework entitled scLLMDA, the schematic is shown in Fig 1. Our method leverages a DNA-specific pretrained language model to obtain rich, context-aware representations of DNA sequences. These representations are combined with peak accessibility information to learn expressive embeddings for each cell. Furthermore, we construct cell-cell graphs based on embedding similarity and employ graph neural networks to incorporate local and global structure into both the feature learning and domain adaptation processes. By integrating sequence-level semantics and graph-aware domain alignment, our approach achieves more accurate and robust cell type annotation for scATAC-seq datasets. In summary, our contributions are threefold:

- We introduce DNA large language model into the scATAC-seq annotation pipeline to enhance sequence-level feature representation;
- We design a graph-based domain adaptation strategy that captures neighborhood structure during source-target alignment;
- We demonstrate superior performance over existing methods on multiple benchmark datasets, showing strong generalizability across domains.

## Materials and methods

### Benchmark datasets

In this study, we utilized several publicly available single-nucleus ATAC-seq (snATAC-seq, A sequencing platform of the ATAC-seq technique) datasets to comprehensively evaluate the performance of the proposed method. These datasets cover different tissue regions and sequencing platforms. Specifically, MosA1, MosM1, and MosP1 were derived from chromatin accessibility profiling of distinct functional regions within the cerebral cortex [17,18], annotated based on the GRCm38 reference genome, and are available from the GEO database (accession number: GSE126724). The Mouse Brain (10x) dataset was generated using the 10x Genomics platform with mm10 as the reference genome. WholeBrainA and WholeBrainB are atlas-style datasets [19] produced using sciATAC-seq [20] (A sequencing platform of the ATAC-seq technique) and annotated with the mm9 reference genome; they are accessible via the GEO database (accession number: GSE111586).

### Benchmark methods

To evaluate the effectiveness of our proposed approach, we compare it against seven representative baseline methods. scNym [21], scJoint [3], and Cellcano [6] rely solely on gene peak features without incorporating DNA sequence information. For scJoint, we use scATAC-seq data during both the training and testing phases. Cellcano is evaluated using its original settings: when the target cell number in the test set is small, results from the first training round are used; otherwise, results from the second round are applied. In contrast, SANGO [15] integrates both DNA sequence and peak-based features for joint representation learning. annATAC [22] is a language model–based framework for automatic scATAC-seq cell type annotation, leveraging pre-training, fine-tuning, and prediction to achieve accurate labeling. AtacAnnoR [23] is a reference-based scATAC-seq cell type annotation method that employs a two-round strategy to transfer labels from annotated scRNA-seq data and refine predictions using genome-wide chromatin accessibility features. MINGLE [24] is a mutual information–based interpretable framework for scATAC-seq cell type annotation that integrates cellular similarity and topological structure modeling to improve robustness and enable identification of rare or novel cell types.To ensure fairness, all training data are preprocessed following the same pipeline as used in SANGO.

### Problem definition

We consider the task of intra-modality cell type annotation for scATAC-seq data, where both the reference (source domain) and query (target domain) datasets originate from the same modality. Although both datasets are collected using scATAC-seq, they may exhibit significant batch effect due to variations in sequencing platforms(e.g., snATAC-seq, 10x, sciATAC-seq), tissue sources, or experimental settings [25]. These discrepancies can lead to distributional shifts in the feature space between the source and target domains, which pose challenges to direct label transfer and necessitate effective domain adaptation strategies [26]. Formally, let:

- ${𝒟}_{s}=\{(\overset{s}{\underset{i}{x}},\overset{s}{\underset{i}{y}})\overset{N_{s}}{\underset{i=1}{\}}}$ denote the source domain, where $\overset{s}{\underset{i}{x}}$ is the DNA sequence and peak signal representation for cell *i*, and $\overset{s}{\underset{i}{y}}\in \{1,2,\ldots ,C\}$ is the corresponding cell type label.
- ${𝒟}_{t}=\{\overset{t}{\underset{j}{x}}\overset{N_{t}}{\underset{j=1}{\}}}$ denote the target domain, where labels are unavailable and need to be inferred.

**Fig 2:**
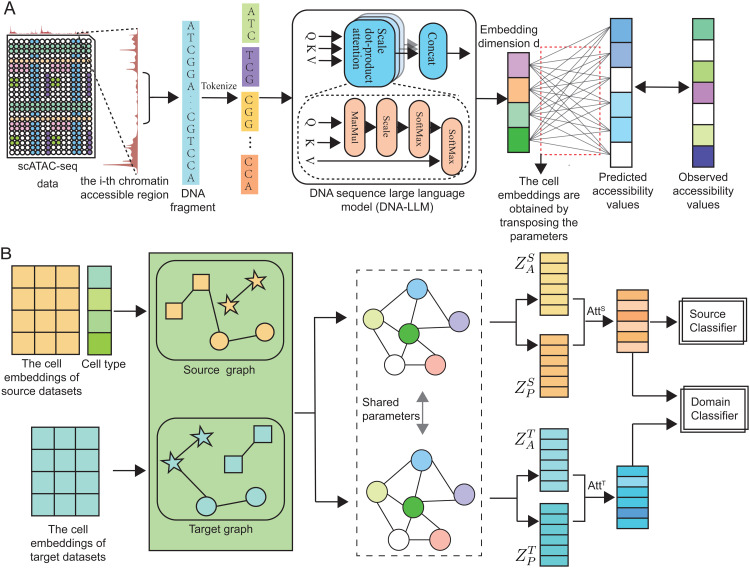
Overview of the proposed scLLMDA framework for cell type annotation from scATAC-seq data. **(A)** *Feature extraction from genomic sequences*: Chromatin-accessible regions are tokenized and encoded using a pretrained DNA language model (DNA-LLM), generating contextualized embeddings that are used to predict accessibility profiles and derive cell embeddings. **(B)** *Cell type annotation via graph domain adaptation*: Source and target cell embeddings are used to construct corresponding graphs. A domain adaptation module with attention mechanisms and shared parameters enables effective knowledge transfer and cell type classification across datasets.

Our objective is to learn a function f:x→y such that for each $\overset{t}{\underset{j}{x}}\in {𝒟}_{t}$, the predicted label $\overset{t}{\underset{j}{\hat{y}}}=f(\overset{t}{\underset{j}{x}})$ is as accurate as possible. To this end, we define a two-stage solution: Step 1: Feature Extraction from Genomic Sequences; Step 2: Cell Type Annotation via Graph Domain Adaptation.The overall framework of our proposed method is illustrated in Fig 2.

### Feature extraction from genomic sequences

To extract biologically meaningful sequence features, we utilize the pretrained DNA language model [27] (i.e., DNABERT-2 [28]) to perform contextualized encoding of each peak sequence. In contrast to conventional convolutional approaches, DNABERT-2 captures intricate regulatory syntax and long-range dependencies, including transcription factor binding patterns and motif co-occurrence. The resulting sequence embeddings integrate both local sequence motifs and higher-order regulatory semantics, thereby offering a comprehensive and biologically enriched feature representation.

Let ${\text{seq}}_{i}$ denote the DNA sequence corresponding to the *i*-th peak in cell *i*. Prior to encoding, each peak sequence is tokenized strictly following the official DNABERT-2 tokenizer. Concretely, we first clean the raw peak sequence by converting it to uppercase and removing non-canonical characters (only A/T/C/G are retained; other symbols such as N are filtered or replaced). We then apply the SentencePiece tokenizer trained with Byte Pair Encoding [29] (BPE) to segment the sequence into a list of variable-length sub-sequence tokens (subwords) according to the learned vocabulary (size 2^12^ = 4096). Next, special tokens required by the Transformer are appended (e.g., a start token and an end/separator token), and the resulting token sequence is mapped to integer IDs to form the model input (optionally with padding/truncation to the maximum length supported by DNABERT-2). These token IDs are finally fed into the Transformer encoder to obtain contextualized embeddings.

The model outputs:


$${𝐳}_{i}=DNABERT({\text{seq}}_{i})\in {\mathbb{R}}^{d^{\prime }},$$ (1)

where ${𝐳}_{i}$ is a high-dimensional embedding of the peak. These peak embeddings $\{{𝐳}_{i}\overset{N_{p}}{\underset{i=1}{\}}}$ are then used to predict the binary accessibility of each peak across *N*_cell_ cells using a fully connected output layer [30]:


$$\hat{𝐘}=\sigma (𝐙{𝐖}_{o}+{𝐛}_{o}),$$ (2)

where $𝐙\in {\mathbb{R}}^{N_{p}\times d}$ is the matrix of all peak embeddings, ${𝐖}_{o}\in {\mathbb{R}}^{d\times N_{\text{cell}}}$ is the learnable weight matrix of the output layer, and σ(·) is the element-wise sigmoid activation. The predicted values $\hat{𝐘}\in [0,1{]}^{N_{p}\times N_{\text{cell}}}$ represent the estimated peak accessibility across all cells. The model is trained by minimizing the binary cross-entropy (BCE) loss between predicted and observed accessibility:


$$\begin{array}{cc} {ℒ}_{\text{BCE}}=-\frac{1}{N_{p}\cdot N_{\text{cell}}}\sum_{i=1}^{N_{p}}\sum_{j=1}^{N_{\text{cell}}}[ & \;y_{ij}\log({\hat{y}}_{ij})\\ & +(1-y_{ij})\log(1-{\hat{y}}_{ij})]. \end{array}$$ (3)

After training, we extract the column-wise weight vectors from ${𝐖}_{o}$ as the cell embeddings for the source and target datasets, respectively. Each column ${𝐰}_{j}\in {\mathbb{R}}^{d}$ of ${𝐖}_{o}$ serves as the *d*-dimensional representation of cell *j*.

### Cell type annotation via graph domain adaptation

#### Graph construction.

Given the learned embeddings of all cells by the Step 1, we construct two *k*-nearest neighbor graphs based on similarity in the embedding space:

- Source domain graph: $G_{s}=({𝒱}_{s},{ℰ}_{s},{𝐗}_{s})$, where ${𝒱}_{s}$ are labeled source cells, ${ℰ}_{s}$ are edges computed using the KNN algorithm, and ${𝐗}_{s}$ is the feature matrix.
- Target domain graph: $G_{t}=({𝒱}_{t},{ℰ}_{t},{𝐗}_{t})$, similarly defined for unlabeled target cells.

#### Capture the local consistency relationship of each graph.

To perform local structural representation learning for both the source graph $G_{s}=({𝒱}_{s},{ℰ}_{s},{𝐗}_{s})$ and the target graph $G_{t}=({𝒱}_{t},{ℰ}_{t},{𝐗}_{t})$, we introduce a customized graph convolutional module, AgConv, built upon CachedGCNConv—a computationally efficient variant of the GCN originally proposed by Kipf and Welling [31]. This design enhances the model’s capacity to capture neighborhood-level interactions while maintaining computational scalability.

The forward propagation rule for AgConv at the *l*-th layer is defined as:


$${𝐇}^{\left(l+1\right)}=\phi \left({\tilde{𝐃}}^{-1/2}\tilde{𝐀}{\tilde{𝐃}}^{-1/2}{𝐇}^{\left(l\right)}{𝐖}^{\left(l\right)}+{𝐛}^{\left(l\right)}\right),$$ (4)

where $\tilde{𝐀}=𝐀+𝐈$ is the adjacency matrix augmented with self-loops, and $\tilde{𝐃}$ is the corresponding degree matrix with diagonal entries $\tilde{D}ii=\sum_{j}\tilde{A}ij$. Here, ${𝐇}^{\left(l\right)}$ denotes the input cell features at layer *l*, while ${𝐖}^{\left(l\right)}$ and ${𝐛}^{\left(l\right)}$ are the learnable weight matrix and bias vector, respectively. The function ϕ(·) denotes a ReLU activation function.

AgConv is applied independently to the source and target graphs, yielding the localized feature representations $\overset{S}{\underset{A}{𝐙}}$ and $\overset{T}{\underset{A}{𝐙}}$ for the source and target domains, respectively.

#### Capture the global consistency relationship of each graph.

To overcome the representational limitations of relying solely on first-order neighborhood information and to improve the model’s capacity to capture higher-order structural dependencies [32], we introduce a Positive Pointwise Mutual Information (PPMI)–based Graph Convolution module, referred to as PgConv. This mechanism is applied to both the source domain graph $G_{s}=(V_{s},E_{s},X_{s})$ and the target domain graph $G_{t}=(V_{t},E_{t},X_{t})$.

Given an input graph *G* = (*V*, *E*, *X*), where *V* denotes the set of nodes, E⊆V×V the set of edges, and *X* the cell feature matrix, we perform random walks [33] of a maximum length *L* from each node u∈V. For every other node v∈V visited during these walks, we record the visitation count as *C*(*u*, *v*). This leads to the empirical conditional probability of observing node *v* given node *u*, defined as:


$$P(v|u)=\frac{C(u,v)}{\sum_{v^{\prime }\in V}C(u,v^{\prime })}$$ (5)

This probability reflects how frequently node *v* appears in the local neighborhood of node *u* based on the random walk trajectory.

To quantify the global occurrence of node *v* across all cell contexts [34], we define the marginal probability as:


$$P(v)=\sum_{u\in V}P(v|u)$$ (6)

The Pointwise Mutual Information (PMI) for each cell pair (*u*, *v*) is computed as:


$$\text{PMI}(u,v)=\log\left(\frac{P(v|u)}{P(v)}\right)$$ (7)

To retain only statistically significant node associations, we define the PPMI as:


$$\text{PPMI}(u,v)=\max\left(0,\log\left(\frac{P(v|u)}{P(v)}\cdot \frac{\left|V\right|}{L}\right)\right)$$ (8)

where |*V*| denotes the number of cells in the graph, and *L* is the maximum walk length, serving as a normalization factor. Based on these scores, a new weighted edge set is formed:


$$E^{\prime }=\left\{(u,v,w_{uv})|w_{uv}=\text{PPMI}(u,v),\;w_{uv}>0\right\}$$ (9)

This process is applied to both the source and target graphs, yielding the corresponding PPMI-weighted adjacency matrices $P_{s}$ and $P_{t}$, respectively.

Given a PPMI matrix *P* (either $P_{s}$ or $P_{t}$), we perform graph convolution using the following propagation rule:


$$H^{\left(l+1\right)}=\phi \left({\tilde{D}}^{-1/2}P{\tilde{D}}^{-1/2}H^{\left(l\right)}W^{\left(l\right)}+b^{\left(l\right)}\right)$$ (10)

Here, $\tilde{D}$ denotes the degree matrix of *P*, *W*^(l)^ and *b*^(l)^ are the learnable weights and biases of the *l* -th layer (shared with the AgConv modu*l*e), and ϕ(·) represents a non-linear activation function, such as ReLU. This operation yields the global node embeddings for the source and target graphs, denoted by $\overset{S}{\underset{P}{Z}}$ and $\overset{T}{\underset{P}{Z}}$, respectively.

#### Feature fusion via attention.

To integrate both local and global information of cells within the graph, we design an attention-based feature fusion module [35,36], which is applied independently to the source domain graph and the target domain graph. This module unifies the features extracted from AgConv (local structure) and PgConv (global semantics) and performs weighted fusion to obtain more expressive cell representations.

Specifically, for a given graph *G* = (*V*, *E*, *X*), let $Z_{A}$ denote the node features extracted via AgConv and $Z_{P}$ denote the features from PgConv based on PPMI encoding.

First, we concatenate the two feature matrices:


$$H=\text{stack}(Z_{A},Z_{P})$$ (11)

Next, we introduce three shared linear projections to transform the stacked features into the query, key, and value matrices:


$$Q=HW_{Q},\;K=HW_{K},\;V=HW_{V}$$ (12)

We then compute attention weights across different feature types and obtain the attention-enhanced representation:


$$H_{\text{att}}=\text{softmax}\left(\frac{QK^{⊤}}{\sqrt{d}}\right)V$$ (13)

Finally, we aggregate the representations to produce the fused cell features:


$$Z=\text{Aggregate}(H_{\text{att}})$$ (14)

This fusion procedure is applied separately to both source and target graphs, i.e., to $\overset{S}{\underset{A}{Z}},\overset{S}{\underset{P}{Z}}$ and $\overset{T}{\underset{A}{Z}},\overset{T}{\underset{P}{Z}}$, resulting in the final fused representations $Z^{S}$ and $Z^{T}$, respectively.

### Loss function

In our model, the training objective jointly considers classification accuracy on the source domain and domain alignment between the source and target domains. Let $Z^{S}$ and $Z^{T}$ denote the learned cell embeddings for the source and target domain graphs, respectively.

The overall loss function is defined as:


$$ℒ={ℒ}_{\text{cls}}+\lambda \cdot {ℒ}_{\text{grl}}$$ (15)

where ${ℒ}_{\text{cls}}$ is the supervised classification loss on the source domain, ${ℒ}_{\text{grl}}$ is the domain adversarial loss [37] based on a gradient reversal mechanism, and λ is a hyperparameter balancing the two terms.

The classification loss is defined as the average multi-class negative log-likelihood [38] over all labeled source domain nodes:


$${ℒ}_{\text{cls}}=-\frac{1}{V_{s}}\sum_{i=1}^{V_{s}}\sum_{c=1}^{C}𝕀(\overset{S}{\underset{i}{y}}=c)\cdot \log\overset{S}{\underset{c}{p}}(i)$$ (16)

where $V_{s}$ is the number of source domain nodes, *C* is the number of classes, $\overset{S}{\underset{i}{y}}$ is the ground truth label of the *i* -th source node, $\overset{S}{\underset{c}{p}}(i)$ is the predicted probability that node *i* belongs to class *c*, and 𝕀(·) is the indicator function that returns 1 if the condition holds and 0 otherwise.

To align the source and target domains, we introduce a gradient reversal layer and train a domain discriminator $f_{\text{dom}}(\cdot )$ that encourages the node embeddings to be domain-invariant. The domain adversarial loss is defined as a binary cross-entropy loss over both source and target nodes:


$${ℒ}_{\text{grl}}=-\frac{1}{V_{s}}\sum_{i=1}^{V_{s}}\log\overset{S}{\underset{0}{p}}(i)-\frac{1}{V_{t}}\sum_{j=1}^{V_{t}}\log\overset{T}{\underset{1}{p}}(j)$$ (17)

where $\overset{S}{\underset{0}{p}}(i)$ is the predicted probability that the domain discriminator classifies source node *i* as “source,” and $\overset{T}{\underset{1}{p}}(j)$ is the predicted probability that target node *j* is classified as “target,” with $V_{t}$ being the number of target domain nodes.

### Parameter settings

During the cell representation learning phase, we use a MLP with hidden dimensions of 512, 256, and 128, respectively. The initial learning rate is set to 1e-3, and the model is trained for 400 epochs. For the graph domain adaptation stage, all nodes from both the source and target graphs are jointly fed into the model for training. A unified learning rate of 3e-3 is used in this stage, and the GCNs for both source and target domains consist of two hidden layers (*L* = 2) with a network architecture of 100–16. The number of training epochs varies depending on the source graph: 55 epochs when the source graph is MosP1, MosA1, or MosM1; 122 epochs for Mouse Brain (10x); and 22 epochs for WholeBrainA or WholeBrainB. The only exception is when WholeBrainB is used as the source and MosM1 as the target, where training is conducted for 138 epochs.

## Results

### Intra-platform cell type annotation

As shown in Table 1, all evaluated methods exhibit strong performance on the intra-platform cell type annotation tasks, such as MosA1 → MosM1, MosM1 → MosP1, and MosA1 → MosP1, where the reference and query datasets are derived from the same sequencing platform and share similar experimental conditions. In these cases, the performance gap between different methods is marginal (e.g., most methods achieve accuracy above 0.95), indicating that batch effects and distributional shifts are relatively mild in intra-platform scenarios. This also suggests that simpler models relying primarily on peak-based features may suffice when the domain discrepancy is small.

**Table 1:** Performance comparison (Accuracy and F1-score) of intra-platform cell type annotation.

Method	R: WholeBrainA Q: WholeBrainB		R: WholeBrainB Q: WholeBrainA		R: MosA1 Q: MosM1		R: MosM1 Q: MosA1	
	Acc	F1	Acc	F1	Acc	F1	Acc	F1
scLLMDA	0.9351	0.7673	0.9184	0.8277	0.9682	0.9618	0.9780	0.9757
SANGO	0.9358	0.7899	0.9150	**0.8625**	**0.9780**	**0.9750**	0.9763	0.9746
scNym	0.9125	0.8152	0.8970	0.7776	0.9522	0.9434	0.9637	0.9560
scJoint	**0.9425**	**0.8414**	**0.9203**	0.8014	0.9730	0.9682	**0.9794**	**0.9761**
Cellcano	0.8847	0.7261	0.8658	0.7008	0.9207	0.9005	0.9226	0.9063
annATAC	0.8837	0.6942	0.7812	0.4904	0.947	0.9372	0.9650	0.9605
AtacAnnoR	0.8655	0.6124	0.9044	0.832	0.8871	0.8747	0.8767	0.8679
MINGLE	0.9051	0.8294	0.9139	0.7984	0.9493	0.9421	0.9503	0.9525
**Method**	R: MosA1 Q: MosP1		R: MosP1 Q: MosA1		R: MosM1 R: MosP1		Q: MosP1 Q: MosM1	
	Acc	F1	Acc	F1	Acc	F1	Acc	F1
scLLMDA	0.9688	0.9632	**0.9804**	0.9776	0.9713	0.9635	0.9706	0.9590
SANGO	**0.9770**	**0.9777**	0.9773	**0.9791**	**0.9796**	**0.9748**	0.9625	0.9616
scNym	0.9552	0.9523	0.9644	0.9581	0.9587	0.9456	0.9583	0.9453
scJoint	0.9659	0.9684	0.9738	0.9719	0.9765	0.9726	**0.9733**	**0.9662**
Cellcano	0.9231	0.8969	0.9234	0.9056	0.9257	0.8991	0.9247	0.9003
annATAC	0.9266	0.9365	0.9607	0.9549	0.9638	0.9563	0.9456	0.9434
AtacAnnoR	0.8791	0.8325	0.8844	0.8748	0.8876	0.8421	0.8621	0.845
MINGLE	0.9539	0.9468	0.9405	0.9416	0.9341	0.9067	0.9494	0.9476

Although our method incorporates a batch effect correction module specifically designed for cross-platform cell type annotation, it still achieves comparable or even slightly superior annotation accuracy on intra-platform datasets.

### Cross-platform cell type annotation

To investigate the robustness of cell type annotation across sequencing platforms, we conducted comprehensive evaluations involving both snATAC-seq and sciATAC-seq datasets. As shown in Table 2, we performed bi-directional transfer experiments between snATAC-seq datasets (MosA1, MosM1, MosP1) and sciATAC-seq datasets (WholeBrainA, WholeBrainB). Table 3 further complements the analysis by including the 10x Genomics-based MouseBrain(10x) dataset as the reference or query in cross-platform scenarios.

**Table 2:** Cell type annotation between snATAC-seq and sciATAC-seq platforms.

Method	Ref: MosA1 Q: WholeBrainA		R: WholeBrainA Q: MosA1		R: MosP1 Q: WholeBrainA		R: WholeBrainA Q: MosP1		R: MosM1 Q: WholeBrainB		R: WholeBrainB Q: MosM1	
	Acc	F1	Acc	F1	Acc	F1	Acc	F1	Acc	F1	Acc	F1
SANGO	0.6508	0.5868	0.6964	0.2981	0.7065	0.6077	0.6062	0.2940	0.6693	0.6035	0.6525	0.3034
scNym	0.6817	0.5843	0.7471	0.3108	0.6937	0.5986	0.6643	0.2932	0.6739	0.6075	0.5877	0.3101
scJoint	0.6452	0.5678	0.7569	0.3403	0.5572	0.5237	**0.7081**	0.3494	0.6574	0.5878	0.7422	0.4118
Cellcano	0.6416	0.5388	0.5335	0.3035	0.6409	0.5350	0.4686	0.2952	0.5970	0.4913	0.4173	0.2803
annATAC	0.6342	0.5578	0.6654	0.2966	0.6320	0.5127	0.5228	0.1938	0.6010	0.5252	0.390	0.2052
AtacAnnoR	0.4523	0.3544	0.5442	0.2317	0.4658	0.371	0.3878	0.2333	0.6648	0.5956	0.2827	0.2025
MINGLE	0.7143	0.6256	0.7095	0.32	0.7097	0.6243	0.6459	0.2973	0.6665	0.6022	0.7033	0.3756
scLLMDA	**0.7228**	**0.6525**	**0.7691**	**0.3993**	**0.7114**	**0.6253**	0.7044	**0.3630**	**0.7012**	**0.6490**	**0.7583**	**0.4580**

**Table 3:** Cell type annotation between MouseBrain(10x) and sciATAC-seq platforms.

Method	R:MouseBrain(10x)	R: WholeBrainA	R: MouseBrain(10x)	R: WholeBrainB
	Q: WholeBrainA	Q: MouseBrain(10x)	Q: WholeBrainB	Q: MouseBrain(10x)
	Acc / F1	Acc / F1	Acc / F1	Acc / F1
SANGO	0.7309 / 0.6307	0.7019 / 0.3614	0.7006 / 0.6270	0.7003 / 0.3601
scNym	0.6636 / 0.5466	0.7568 / 0.3661	0.6631 / 0.5754	**0.7794** / 0.3712
scJoint	0.7097 / 0.6154	0.7453 / 0.3541	0.6813 / 0.6131	0.7611 / 0.3576
Cellcano	0.6324 / 0.5245	0.6978 / 0.3705	0.6050 / 0.5094	0.6801 / 0.3635
annATAC	0.6735/ 0.5392	0.7527/ 0.3934	0.6659 / 0.5690	0.7469 / 0.3349
AtacAnnoR	0.6648 / 0.5956	0.5942 / 0.2813	0.5885 / 0.4776	0.3782 / 0.2837
MINGLE	0.7256 / 0.6255	0.721 / 0.4069	0.6938 / 0.6196	0.7696 / 0.3999
scLLMDA	**0.7423** / **0.6616**	**0.7606** / **0.4141**	**0.7359** / **0.6703**	0.7423 / **0.4249**

Across both tables, our proposed method, scLLMDA, consistently achieves superior performance compared to all baseline methods in terms of both accuracy and macro-F1 score, with the exceptions of the WholeBrainB → MouseBrain(10x) and WholeBrainA → MosP1 transfer scenarios. Notably, scLLMDA achieves the best performance in most evaluated directions, demonstrating strong generalization when transferring annotations between platforms with differing technical properties and cell coverage. For example, in the challenging transfer from WholeBrainA to MosA1 (Table 2), scLLMDA achieves an accuracy of 0.7691 and a macro-F1 of 0.3993, outperforming scJoint and scNym by substantial margins. Similar trends are observed in transfers involving MouseBrain(10x), where scLLMDA obtains the best F1 score (0.4249) in the WholeBrainB → MouseBrain(10x) direction (Table 3).

These results highlight two key observations. First, peak-only methods such as scNym, scJoint, and Cellcano show moderate performance but struggle to generalize across technologies, particularly in reverse transfer settings. Second, sequence-aware models like SANGO offer improvements, but integrating DNA sequence features with domain adaptation (as done in scLLMDA) further boosts performance significantly.

In summary, scLLMDA demonstrates strong cross-platform transferability, particularly in scenarios involving heterogeneous sequencing protocols (snATAC-seq vs. sciATAC-seq) and variable cell type granularity. This validates the advantage of combining local chromatin accessibility patterns with global regulatory context via sequence-based representation learning.

### UMAP visualization comparison across methods

**Fig 3:**
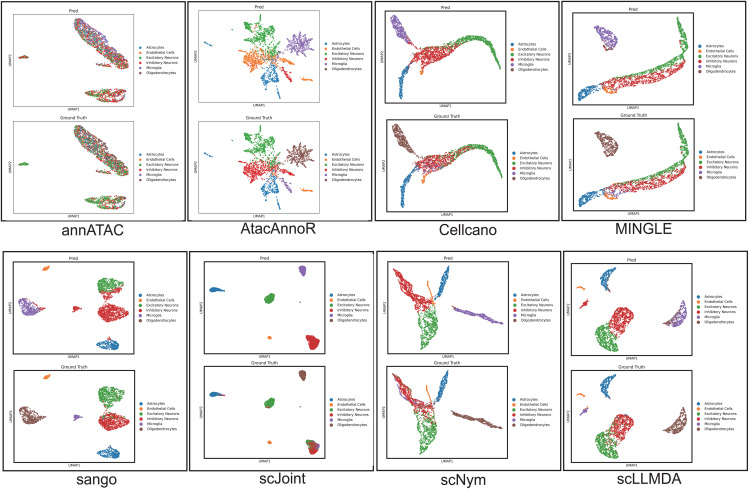
UMAP visualization comparison of cell type annotation results under the MosA1 → WholeBrainA transfer task. Predicted labels (top row) and ground-truth labels (bottom row) are shown for each method, including annATAC, AtacAnnoR, Cellcano, MINGLE, SANGO, scJoint, scNym, and scLLMDA.

To further provide an intuitive comparison of annotation quality across methods, we visualize the predicted labels and corresponding ground-truth labels using UMAP embeddings under the MosA1 → WholeBrainA transfer task. The visualizations of annATAC, AtacAnnoR, Cellcano, MINGLE, SANGO, scJoint, scNym, and scLLMDA are shown in Fig 3.

For annATAC, it is important to note that the model does not directly output cell-level embeddings. Instead, annATAC produces sequence-level representations with shape (*N*, *L*, 200), where *N* denotes the number of cells and *L* corresponds to the peak dimension. Since no officially defined cell embedding is provided, we obtained approximate cell-level representations by averaging along the peak dimension, yielding (*N*, 200) features for UMAP visualization. This post-processing step may partially affect the geometric structure of annATAC embeddings and explains the atypical visualization pattern observed in Fig 3.

From the UMAP comparisons, several observations can be made. First, most baseline methods exhibit notable mixing between cell populations, indicating limited separability under cross-platform transfer. In particular, methods such as scNym, scJoint, Cellcano, MINGLE, and AtacAnnoR show substantial overlap among neuronal subtypes and glial populations, reflecting challenges in capturing discriminative regulatory patterns across technologies. Second, sequence-aware methods such as SANGO demonstrate improved clustering structure but still display fragmented or dispersed clusters for certain cell types.

A particularly challenging case is the Oligodendrocyte population. As shown in the prediction panels, nearly all baseline methods fail to recover a coherent cluster for this cell type, resulting in extremely low recognition rates. In contrast, scLLMDA successfully identifies a subset of Oligodendrocyte cells and forms a partially separable cluster consistent with the ground-truth distribution. This improvement highlights the benefit of incorporating sequence-informed regulatory representations together with domain adaptation to capture subtle chromatin accessibility signatures.

Overall, the UMAP visualization corroborates the quantitative results by demonstrating that scLLMDA produces more structured and biologically meaningful embedding spaces, leading to improved cross-platform cell type discrimination.

### Computational efficiency and scalability

Scalability is a major concern for single-cell omics methods, especially under cross-dataset annotation scenarios. To evaluate scalability, we conducted systematic computational efficiency benchmarks in terms of running time and GPU memory usage on two representative cross-dataset annotation tasks, namely MosA1 → WholeBrainA and MouseBrain(10x)→WholeBrainA. We compared scLLMDA with several representative baselines, including sequence-based and expression-based methods. The results are summarized in Table 4.

**Table 4:** Computational efficiency benchmarks (time and memory) on two cross-dataset annotation tasks. For CPU-only methods, we report “CPU” in the memory column.

MosA1 → WholeBrainA			MouseBrain(10x)→WholeBrainA		
Method	Time	Memory	Method	Time	Memory
SANGO	126min20s+50s = 127min10s	1656MiB	SANGO	116min12s+43s = 116min55s	1656MiB
scNym	8min5s+11s = 8min16s	1094MiB	scNym	5min35s+10s = 5min45s	1094MiB
scJoint	4min24s	2742MiB	scJoint	4min11s	2742MiB
Cellcano	8min16s+1min5s = 9min21s	CPU	Cellcano	4min19s+1min7s = 5min26s	CPU
scLLMDA	15min19s+37min40s + 38s = 53min37s	1186MiB	scLLMDA	15min42s+23min4s + 33s = 39min19s	1186MiB
annATAC	116min21s+56s = 117min17s	22528MiB	annATAC	50min51s+58s = 51min49s	22728MiB
AtacAnnoR	3min36s	CPU	AtacAnnoR	6min56s	CPU
MINGLE	1min6s	13352MiB	MINGLE	1min2s	13998MiB

For scLLMDA, the overall pipeline consists of three stages: (1) DNABERT-2 feature extraction, (2) cell embedding learning, and (3) graph construction with domain adaptation. On the MosA1 → WholeBrainA task, the total running time is 53min37s, with 15min19s, 37min40s, and 38s for the three stages, respectively. The corresponding GPU memory consumption is 990MiB, 954MiB, and 1186MiB (peak ~1.2GB). Similar behavior is observed on the MouseBrain(10x)→WholeBrainA task, with a total running time of 39min19s and a comparable peak memory footprint.

Compared to the sequence modeling baseline SANGO, scLLMDA achieves substantially lower computational overhead while providing improved annotation performance. For example, on the MosA1 → WholeBrainA task, SANGO requires 127min10s and 1656MiB, whereas scLLMDA completes in 53min37s with ~1.2GB peak GPU memory, reducing runtime by over 50%. Although scLLMDA is slower than some expression-based methods (e.g., scNym, scJoint) due to the additional DNABERT-2 sequence modeling, it remains significantly more memory-efficient than methods such as annATAC (around 22GB). Overall, the benchmarks demonstrate that scLLMDA maintains a favorable balance between efficiency and accuracy among sequence-informed approaches, indicating good scalability in practical GPU settings.

### Ablation study

#### Sensitivity analysis of the balancing hyperparameter λ.

**Fig 4:**
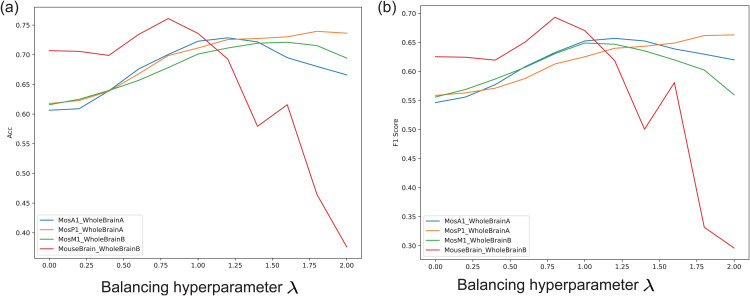
Effect of the balancing hyperparameter λ on cross-platform annotation performance of scLLMDA. **(a)** Accuracy; **(b)** F1-score.

We selected four representative cross-platform annotation tasks to evaluate the sensitivity of scLLMDA to the balancing hyperparameter λ. As shown in Fig 4, with the gradual increase of λ, the model’s performance in terms of accuracy and F1 score generally exhibits a rising-then-falling trend, with the optimal performance typically occurring around λ≈1.0. This observation suggests that introducing a moderate domain adversarial signal effectively enhances the model’s adaptability to distributional shifts between platforms. However, setting λ too large introduces an overly strong adversarial signal, which suppresses the learning of task-discriminative features in the main classification objective and leads to degraded overall performance. This degradation is especially prominent in challenging scenarios with significant platform discrepancies, such as *MouseBrain* → *WholeBrainB*. Therefore, careful tuning of λ is crucial for achieving accurate and robust transfer annotation. Based on multiple experiments, we recommend setting λ within the range of 0.9 to 1.1, where scLLMDA strikes a good balance between accuracy and stability, demonstrating strong generalization and cross-platform transfer performance.

### Effectiveness of GDA module

To evaluate the effectiveness of the proposed GDA module in the scLLMDA framework, we conducted an ablation study comparing two variants: (i) DNABERT+GDA, the full pipeline where cell embeddings extracted in the first stage are used for annotation through the GDA module; and (ii) DNABERT+KNN, where the GDA module is replaced with a *k*-nearest neighbor classifier (*k* = 6), serving as a baseline.

**Fig 5:**
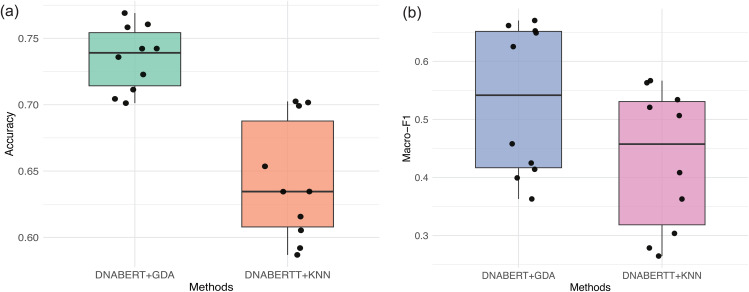
Ablation study comparing DNABERT+GDA and DNABERT+KNN across 10 cross-dataset annotation tasks. The boxplots display distributions of **(a)** Accuracy and **(b)** Macro-F1 score.

The experiment is performed across 10 transfer tasks involving different sequencing platforms (e.g., MosA1, MouseBrain(10x), WholeBrainA/B), with performance measured by accuracy and macro-F1 score. As shown in Fig 5, DNABERT+GDA consistently outperforms DNABERT+KNN across both metrics. The boxplots illustrate that DNABERT+GDA yields higher median accuracy and macro-F1, while black dots represent per-task results, confirming the robustness of GDA across diverse settings.

In particular, GDA demonstrates significant advantages in more challenging scenarios involving domain shifts, suggesting its ability to align feature distributions and enhance the recognition of minority or ambiguous cell types. This ablation study highlights that domain adversarial training is crucial for improving generalization in cell type annotation tasks based on scATAC-seq data.

## Discussion

In this study, we proposed scLLMDA, a novel framework that combines DNA language model and graph-based domain adaptation for accurate scATAC-seq cell type annotation. Experimental results show that scLLMDA consistently outperforms existing methods across both intra- and cross-platform settings. The key strengths of our method lie in its ability to capture rich sequence semantics and structural relationships between cells. By integrating contextual DNA embeddings with local and global graph information, scLLMDA effectively mitigates domain shifts and improves annotation robustness.

Despite its effectiveness, our framework has limitations. First, the computational cost of using large-scale pretrained DNA language models can be non-trivial [39], especially for datasets with hundreds of thousands of peaks. Efficient adaptation or compression of such models for scATAC-seq is a promising direction. Second, while we consider both local and global graph structure, current edge construction is based purely on similarity in embedding space. Incorporating biological priors—such as known enhancer-promoter interactions or transcription factor binding networks [40]—may further improve interpretability and performance.

Future work may also extend scLLMDA to multi-modal integration, where chromatin accessibility is combined with transcriptomic or epigenomic profiles [41] at the single-cell level. Additionally, applying scLLMDA to disease-related datasets or clinical samples could provide insight into pathological regulatory programs [42,43] and cell-type perturbations.

## Conclusion

We presented scLLMDA, a framework that combines DNA language models with graph-based domain adaptation for accurate scATAC-seq cell type annotation. By capturing sequence semantics and cell–cell structural relationships, scLLMDA achieves robust performance across datasets. Future work will focus on improving efficiency, integrating biological priors, and extending the framework to multi-modal and clinical applications.
